# Electromagnetic levitation containerless processing of metallic materials in microgravity: rapid solidification

**DOI:** 10.1038/s41526-023-00310-2

**Published:** 2023-08-15

**Authors:** D. M. Matson, L. Battezzati, P. K. Galenko, Ch.-A. Gandin, A. K. Gangopadhyay, H. Henein, K. F. Kelton, M. Kolbe, J. Valloton, S. C. Vogel, T. Volkmann

**Affiliations:** 1 Department of Mechanical Engineering, Tufts University, Medford, MA 02155 USA; 2 Dipartimento di Chimica e Centro NIS, Università di Torino, Via P, Giuria 7, 10125 Torino, Italy; 3 Otto-Schott-Institut für Materialforschung, Friedrich Schiller Universität Jena, Jena, Germany; 4 MINES Paris, PSL University, CEMEF UMR CNRS 7635, CS10207, 06904 Sophia Antipolis, France; 5 Department of Physics and the Institute of Materials Science & Engineering, Washington University, St. Louis, MO 63130-4899 USA; 6 Department of Chemical and Materials Engineering, University of Alberta, Edmonton, AB T6G 2G6 Canada; 7 Institut für Materialphysik im Weltraum, Deutsches Zentrum für Luft- und Raumfahrt (DLR), 51170 Köln, Germany; 8 Materials Science and Technology Division, Los Alamos National Laboratory, Los Alamos, NM 87545 USA

**Keywords:** Materials science, Physics

## Abstract

Space levitation processing allows researchers to conduct benchmark tests in an effort to understand the physical phenomena involved in rapid solidification processing, including alloy thermodynamics, nucleation and growth, heat and mass transfer, solid/liquid interface dynamics, macro- and microstructural evolution, and defect formation. Supported by ground-based investigations, a major thrust is to develop and refine robust computational tools based on theoretical and applied approaches. This work is accomplished in conjunction with experiments designed for precise model validation with application to a broad range of industrial processes.

## Introduction

The quantitative and predictive modeling of liquid-state manufacturing processes is now a cornerstone of current engineering projects, starting from alloy selection to solidification-process design, the evolution of the solidification macro- and microstructure, and subsequent thermomechanical treatments to achieve optimum mechanical properties^1^. This includes not only the processes for semi-finished products and shape castings, but also processes entailing rapid solidification, such as high-pressure die casting, strip casting, welding, atomization, spray forming, and additive manufacturing (3D printing). Of particular interest is developing computational tools, in conjunction with model validation testing^2^, to aid in the control of rapid solidification processes.

These issues cover the whole range from optimized solidification techniques to tailoring of the solidification microstructure, combined with optimization of the alloy composition and development of new alloy compositions for a given application—all with an eye to supporting closed-loop recycling. The approach is relevant for all cast products and alloys, ranging from the automotive world, turbine blades for land-based energy production and jet engines, medical implants, large- and small-scale casting, and semi-finished products such as the continuous casting of slabs. In recent years, its high relevance for additive manufacturing processes has also become particularly significant.

Containerless processing, both on ground and in a microgravity environment, is particularly important for conducting benchmark experiments to support solidification physics research topics—particularly for investigations targeting evaluation of phase selection and microstructural evolution following rapid solidification from the undercooled melt and subsequent mushy-zone microstructural evolution. For space testing, an electromagnetic levitation facility (ISS-EML) was developed to enable transformative molten metal and rapid solidification experimentation in the microgravity environment of the International Space Station (ISS)^3^.

The ISS-EML facility utilizes a high-frequency current within water-cooled copper tubes to position and inductively heat conductive samples to extremely high temperature without the need for a crucible. Samples may be melted and subsequently solidified through the selection of key processing parameters. Containerless processing may be accomplished either in a vacuum or in an inert gas-shielding environment to limit sample evaporation. When operated in microgravity, the key attributes for space testing include limiting the influence of buoyancy and sedimentation, maintaining a spherical equilibrium sample shape during levitation to simplify the analysis of processing conditions under dynamic stimulation, and accessing controlled convection conditions which span the range from laminar flow to turbulent flow with the ability to select conditions that optimize the scientific return from a particular set of experiment design considerations. Obviously, close collaboration is required with groups conducting thermophysical property measurements to enable researchers to select and control convection to the specified desired levels for a given test.

This article presents a highlighted summary of a selection of key accomplishments from several of the ESA/DLR/NASA projects utilizing the ISS-EML facility to investigate rapid solidification processes. Each subheading of the discussion pertains to individual projects. The aim is to give the reader a snapshot view of some of the successes achieved and a flavor of the breadth of what may be accomplished using space levitation processing techniques.

## Recent achievements

Space research on rapid solidification focuses on key topics, which include: the structure of metallic melts, the influence of convection on nucleation, the influence of convection on metastable phase selection, growth competition kinetics, atomization, evaluation of the solidification path for multicomponent systems, anomalous growth kinetics, directional solidification, grain refinement, and demixing of liquids and solids. In the following sections, analyses of the experimental result and subsequent modeling highlights from several of the ISS-EML projects are presented.

### Quasicrystalline undercooled alloys for space investigation (QUASI)

The QUASI program seeks to define the relation between dynamics and structure in metallic liquids. Experiments are focused on the measurement of thermophysical properties of supercooled melts, which will be correlated with terrestrial x-ray liquid structure studies. A major focus is on the influence of convection transformation kinetics from liquid to metastable and stable solid phases.

When cooled fast enough to below the glass transition temperature, *T*_g_, any liquid can bypass crystallization and become a metastable glassy solid. An improved understanding of the evolution of short- and medium-range structural order with temperature in liquids and glasses and the accompanying changes in the atomic dynamics is the main goal of this group, using terrestrial and microgravity experiments. The important dynamical properties are viscosity, *η*, and the structural relaxation time, *τ*. The viscosity changes by over sixteen orders of magnitude with only a few hundred degrees change in temperature, from the melting (or liquidus) temperature, *T*_L_, to *T*_g_. As first suggested by Angell^4^, the rate of this change near ${T}_{g}$ is very different for different liquids, which can be expressed in terms of the *fragility* of the liquid. In a *strong* liquid, the temperature dependence of the viscosity is nearly Arrhenius over the entire temperature range, i.e., $\eta \,{\propto }\,{{\exp }}(E/{k}_{{\rm{B}}}T),$ where *E* is activation energy. As the temperature dependence departs from an Arrhenius one, the liquid becomes more *fragile*. The fragility is generally expressed by the fragility index,

1 $$m={\left.\frac{d({\log }_{10}\eta )}{d\left({T}_{{\rm{g}}}/T\right)}\right|}_{{T}_{{\rm{g}}}}$$

Fragility has also been identified in the temperature dependence of other physical quantities, such as the entropy^5^, specific heat^6^, mechanical properties of glasses^7^, and the rate of structural ordering in the liquid^8^ This suggests that there must be a deep link between the structure of the liquid and its dynamical behavior^9,10^.

Because of experimental limitations for accessing this metastable phase below ${T}_{{\rm{L}}}$ (rapid crystallization, chemical reactions with containers, and the environment, to name a few), molecular dynamics (MD) investigations of structural/dynamical correlations have become useful. However, such studies often give questionable results near *T*_g_ because of the vastly different relaxation times between the simulations and experiments, although they can be used to study the high-temperature liquid where the relaxation rates are comparable. Based on MD studies of metallic liquids, fluctuations in the local atomic structure (characterized by the time that it takes for a local cluster to change its coordination number by one, *τ*_LC_) can be correlated with dynamical behavior^11–14^. At high temperatures, the lifetime of these local structural fluctuations is too short to have an impact on nearby regions. However, below a material-dependent temperature, *T*_A_, the lifetime becomes sufficiently long that local fluctuations can influence nearby regions, leading to cooperativity (where many atoms are acting together, almost as a unit). The MD simulations indicate that above *T*_A,_ *τ*_LC_ is equal to the Maxwell characteristic time for viscous flow, ${\tau }_{{\rm{M}}}=\eta /{G}_{{\infty }},$ where ${G}_{{\infty }}$ is the high-frequency shear modulus This suggests that the shear viscosity is governed by single atom excitations above ${T}_{{\rm{A}}}$, giving an Arrhenius temperature dependence for the viscosity and an activation energy that is related to the effective bond strength. As the temperature is lowered below *T*_A_, *τ*_M_ becomes increasingly larger than *τ*_LC_, indicating that viscous flow becomes increasingly more cooperative. Viewing this as an Arrhenius relation for the viscosity, the increasing cooperativity causes the activation energy to become increasingly larger with decreasing temperature. An example of the results on the viscosity between *T*_A_ and *T*_g_ can be found elsewhere^15^. Finally, it should be noted that experiments and MD simulations indicate a correlation between *T*_A_ and the glass transition temperature, *T*_g_,^11,15^.

While the crossover behavior in the viscosity has been experimentally observed and the value of *T*_A_ for different metallic liquids has been measured^15^, clear experimental evidence for a structural connection has been difficult to obtain. X-ray and neutron diffraction are normally used to study the crystalline order in solids from the positions and intensities of the Bragg peaks. In the absence of long-range order in liquids and glasses, the sharp Bragg peaks become very broad and diffuse. Even then, such data can be used to study the short-(atomic scale) and medium-range (over many atomic scales) order. However, since the positions and heights of the diffuse peaks in the structure factor, *S*(*q*) (where q is the momentum transfer, related to the scattering angle), changes little with temperature compared to the 16 orders of magnitude change in viscosity, it is difficult to correlate the liquid dynamics with the structure. A small change in the intensity of a shoulder on the low-*q* side of the 2^nd^ peak of *S*(*q*) as a function of temperature has been found to correlate with *T*_A_^16^, but the change in the intensity of the shoulder occurs at a lower temperature. The weak scattering of the X-rays from the liquid structure could account for this difference, since the structural changes would need to be large to be observed. The electrical resistivity of a metallic liquid is known to be proportional to the integral of the structure factor^17^ It is well-known that electrons scatter more strongly from condensed phases than X-rays. This and a much higher precision in measurements of the electrical resistivity, ${\rho },$ than is possible for $S\left(q\right)$ measurements suggested that the electrical resistivity could reveal the structure/dynamics correlation. Measurements were made on Zr_64_Ni_36_ and Cu_50_Zr_50_ liquids using the EML facility located on the International Space Station. The lack of a container enabled sample contamination to be minimized and facilitated measurements deep into the supercooled metastable liquid below ${T}_{{\rm{L}}}$^18^, as mentioned in the introduction.

**Fig. 1:**
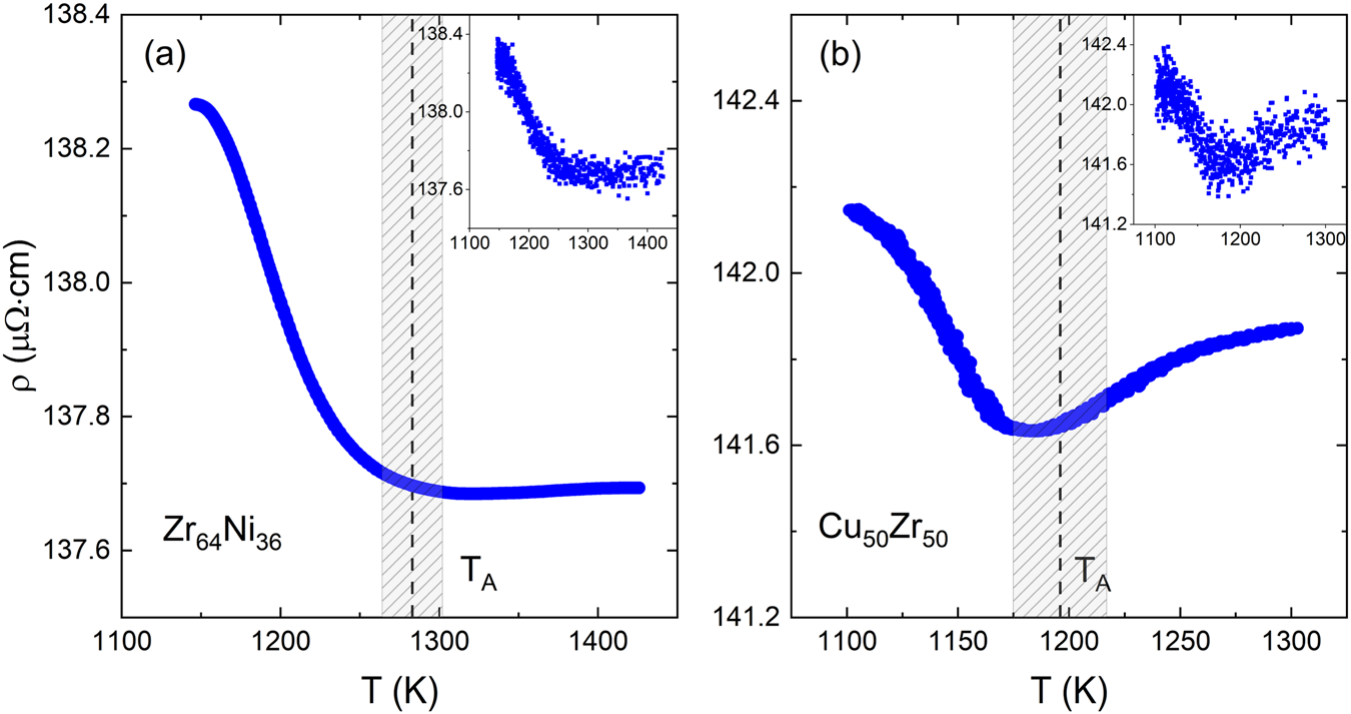
The electrical resistivity, smoothed by 200-point averaging, as a function of liquid temperature near saturation, at or above T_A_, for two binary systems. **a** Zr64Ni36 and **b** Cu50Zr50. The shaded regions represent the uncertainties in *T*_A_. The original unsmoothed data were shown in the insets. The data were reprinted with permission from ref. ^18^. Copyright (2019) by the American Physical Society.

As shown in Fig. 1, the temperature dependence of *ρ* suddenly increases below and near *T*_A_, determined from the viscosity measurements (marked by the dashed vertical line), providing the strongest evidence to date for a change in structure that is correlated with the change in viscosity. Additionally, a surprising feature is observed that is not predicted by any existing theory of the resistivity of metallic liquids—a saturation or near saturation of the resistivity above *T*_A_. While a saturation in the resistivity is known for most solids at very low cryogenic temperatures due to a vanishing of excitations that can scatter the electrons, this saturation is observed at very high temperatures where there should be an abundance of excitations. A possible explanation is that below *T*_A_ the lifetime of the local liquid structure is much longer than the electron scattering times, so the supercooled liquid structure appears to be static with respect to the electrons. The resistivity changes in this temperature range are then largely due to electron/high-frequency phonon scattering. It is noteworthy that the conventional notion of phonons in solids is not applicable in liquids since the shear viscosity of liquids is very small at high temperatures. However, since the high-frequency shear modulus is finite, as was suggested by Frenkel^19^, high-frequency phonons exist in liquids with a characteristic frequency of $1/{\tau }_{{\rm{M}}}.$ With increasing temperature, both the length scales and the structural lifetimes of the dynamically evolving local clusters decrease^11,13,14^. Near *T*_A_, the mean electron scattering time becomes of order picoseconds, comparable with the structural relaxation times determined in our recent measurements of the Van Hove function (the time-dependent pair distribution function)^20^. The results presented here then suggest that the lifetimes of the high-frequency phonons in the small solid-like cluster regions become too short to couple with the electrons. This renders the electron-phonon scattering mechanism ineffective, resulting in a near saturation of the electrical resistivity at and above a temperature region where viscous flow also becomes non-cooperative.

Nucleation is most commonly understood within the framework of the Classical Nucleation Theory (CNT)^21^. While there are some issues with the thermodynamic model used in CNT, the kinetic model appears to be correct for stoichiometric crystallization (where the chemical composition of the initial phases are the same)^22,23^. However, that is not true when the chemical compositions of the initial and final phases are different^24^. In that case, the stochastic processes of interfacial attachment (the joining of an atom of the initial phase to the cluster of the new phase) and long-range diffusion (the transport of atoms in the initial phase to the neighborhood of the nucleating cluster) become coupled; the Coupled-Flux Model (CFM) model of nucleation was developed to treat this^25^.

While kinetic Monte-Carlo studies support key predictions of the CFM^26^, only recently were they confirmed experimentally by using the reduced gravitational environment of the ISS to investigate changes in the nucleation rate with stirring^27^. For small levels of stirring, the nucleation rate of a phase with a different composition than the liquid is controlled by the coupled stochastic processes of interfacial attachment and long-range diffusion. The need for long-range diffusion decreases with increasing stirring rates and the CFM predicts that the nucleation rate should increase. There should be little effect of stirring on the nucleation rate of crystal phases that have the same chemical composition as the liquid. To investigate this, maximum undercooling measurements were made as a function of stirring for a Vit 106 liquid that crystallizes to phases with a different chemical composition than that of the liquid, and for Cu_50_Zr_50_ and Ti_39.5_Zr_39.5_Ni_21_ liquids for which the nucleating phases have the same composition. The heating voltage and positioning voltage of the sample are decoupled to some extent in the electromagnetic levitation (EML) facility aboard the ISS (ISS-EML). For these experiments, different levels of stirring were achieved by different combinations of the positioner and heating voltages.

**Fig. 2:**
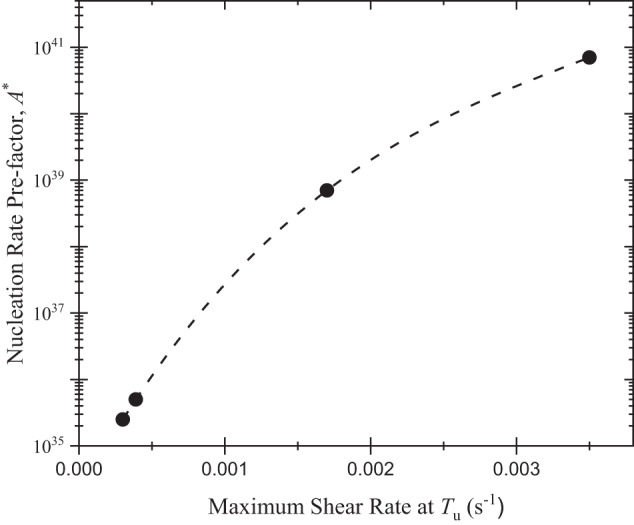
The pre-factor for the nucleation rate as a function of the maximum shear rate at the maximum undercooling temperature of liquid Vit 106 (Zr_57_Cu_15.4_Ni_12.6_Al_10_Nb_5_), processed in vacuum using the ISS-EML. The dashed line is included as a guide to the eye. (Adapted from Fig. 2 in ref. ^27^).

As shown in Fig. 2, the dynamical pre-factor for nucleation, A^*^, increases as the maximum shear rate increases (which scales with the level of stirring) in the Vit 106 liquid. This indicates that the steady-state nucleation rate, *I*^s^, (*I*^s^ = A^*^ exp (-W^*^/*k*_B_*T*), where W^*^ is the critical work of cluster formation), also increases, as predicted from the CFM. As expected, the influence of stirring is small for nucleation in the Cu_50_Zr_50_ and Ti_39.5_Zr_39.5_Ni, liquids^27^; what is surprising is a small decrease in the nucleation rate with increasing shear rate for the Ti_39.5_Zr_39.5_Ni_21_ liquid. Earlier studies have shown that the nucleation of a metastable icosahedral phase is catalyzed by icosahedral ordering in that liquid^28^. The decreasing nucleation rate with increased stirring might, therefore, indicate that stirring disrupts that ordering, although the length scale of that order seems too small to be significantly affected by the stirring rates used. This should be investigated further in future studies.

### Electromagnetic levitation flight support for transient observation of nucleation events (ELFSTONE)

The ELFSTONE project focuses on the influence of melt convection on phase selection and the kinetics of phase transformations.

When we address structural evolution and solidification physics issues, understanding phase selection is one of the pillars of the key space materials science topics^1^. Rapid solidification of Fe-Cr-Ni stainless steels initially involves the formation of metastable ferrite with a subsequent rapid transformation to the equilibrium austenite. Transformation kinetics, phase selection, and microstructural evolution in this industrially important system are strongly influenced by both undercooling and convection. When doing containerless ground electromagnetic levitation (EML) testing, in order to support the sample weight, the positioning force is quite high, which drives significant internal flow—often resulting in turbulent conditions. When doing ground electrostatic levitation (ESL) testing, the positioning force is applied through a static field and there is zero induced stirring. Thus, on ground we can either get too much or too little stirring. We go to space to bridge this gap and investigate the full range of conditions spanning laminar to turbulent flow behavior^29^.

**Fig. 3:**
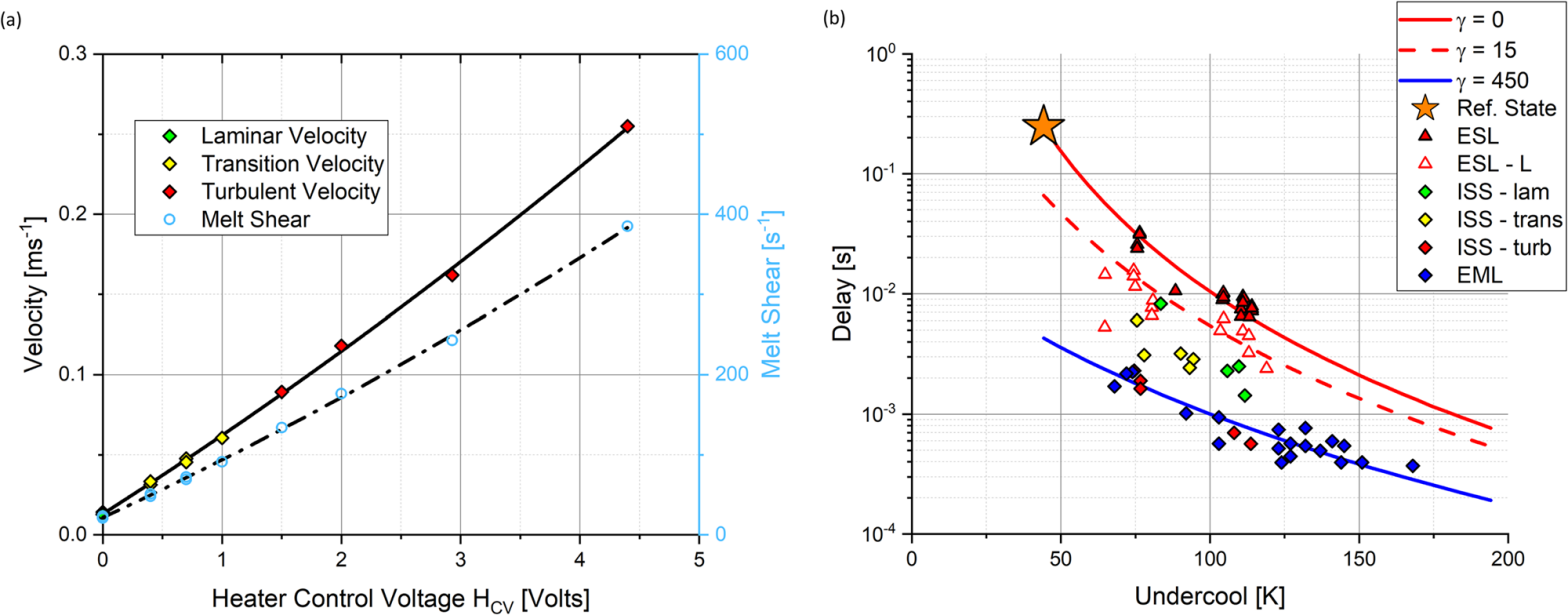
Accessible range of convection conditions for a 60Fe-20Cr-20Ni alloy processed in the ISS-EML facility. Space testing is important because on ground only the extremes in stirring may be investigated, while in space, convection can be controlled over the entire continuum from laminar to turbulent flow. **a** Convection range accessible using the ISS-EML facility by selecting a range of heater control voltage settings; velocity points correspond to space run conditions in laminar (green), transition (yellow), and turbulent (red) flow regimes, while open symbols represent the corresponding melt shear. **b** Delay behavior as a function of undercooling and stirring condition; red/yellow/green colored points correspond to conditions in the previous figure while blue diamonds represent ground EML (significant stirring), open red triangles represent ground ESL(low flow Marangoni convection), and open triangles represent convection-free ESL conditions (zero induced flow).

The ISS-EML facility has as one of its chief attributes the ability to decouple positioning and heating of the sample through superposition of the levitation field^3^. Since thermophysical properties can be readily measured using the ISS-EML^30,31^ a broad range of convection levels are achievable—spanning behavior from laminar to turbulent^32,33^ as seen in Fig. 3a where magnetohydrodynamic (MHD) model predictions are used to guide the selection of processing conditions. In the figure, the maximum velocity at a given temperature, either superheated above the liquidus (T_m_+) or undercooled below the liquidus (T_m_–), is shown as a function of the strength of the heater induction field. Note that a heater limit condition exists where the induction field is too strong to allow the sample to cool. Fig. 3b shows the corresponding incubation delay between the formation of ferrite and austenite as a function of undercooling. The incubation time is the delay between the formation of a metastable phase and subsequent transformation to a stable phase which thus controls localized phase selection and the evolution of defect structures during rapid solidification. Symbols on each part of the Figure represent actual on-orbit conditions. The strength of sample convection may be characterized by evaluating the localized change in the flow of adjacent fluid layers, known as melt shear; the higher the shear, the bigger the difference in relative flow rates and, thus the greater the potential for introducing defects into the growing solid. For each stirring condition, characterized by the observed melt shear, the delay decreases significantly with undercooling. The stirring conditions are indicated by lines where red lines show ground-based ESL tests at zero melt shear, dotted red for ESL with Marangoni convection shear of $\dot{\gamma }$ = 15 s^−1^, blue lines show ground EML tests at $\dot{\gamma }$ = 450 s^−1^, and intermediate points correspond to the space convection settings shown in Fig. 3a. Note that the red turbulent flow space EML data points approach the blue diamond points representing ground EML results where significant convection is observed, and the green laminar flow space data points approach open red triangle ground ESL results characterized by moderately low Marangoni convection. Comparing stirring conditions, as convection increases, the delay also significantly decreases.

This delay behavior is consistent with a growth competition model for phase selection^34–36^ but, as described previously, CNT fails to explain this behavior. A new retained damage model (RDM) was proposed that successfully predicts the influence of both undercooling and convection on delay^37–42^ based on an analogy to hot-working. As in hot-working, the damage is retained by the microstructure in the form of defects with associated free energy that may now drive subsequent transformations—either through recrystallization or transformation to a new phase. There are two sources driving free energy retention. First, the deeper the undercooling, the faster the growth of the metastable, and the more defects and the more free energy may be retained. Here the speed at which the dendrites grow is the key feature driving defect accumulation. Second, the higher the melt shear associated with increased convection, the more defects, and again, the more retained free energy. Here, convection during dendrite growth is the key feature that imparts more damage. Thus, the model shown in Eq. 2 includes two new terms, one relating to undercooling *ΔG*_m_, one relating to convection *ΔG*_c_, and these are added to the CNT calculation of the transformation free energy *ΔG*_s_ that drives nucleation of the second phase. The first two terms are functions of the heat of fusion *ΔH*, the undercooling *ΔT*, and the transformation temperature *T* with subscripts referring to stable *s* and metastable *m* phases. The contribution from convection is based on the melt shear $\dot{\gamma }$ associated with internal recirculating flows calculated using MHD simulation and two empirical constants representing a linear fit Δ_m_ slope and intercept Δ_b_ to the observed data.

2 $$\begin{array}{ll}{\Delta G}_{{\rm{T}}}={\Delta G}_{{\rm{s}}}+{\Delta G}_{{\rm{m}}}+{\Delta G}_{{\rm{c}}} & {\Delta G}_{{\rm{s}}}={\Delta H}_{{\rm{s}}}{\Delta T}_{{\rm{s}}}/{T}_{{\rm{s}}}\\ & {\Delta G}_{{\rm{m}}}={\Delta H}_{{\rm{m}}}{\Delta T}_{{\rm{m}}}/{T}_{{\rm{m}}}\\ & {\Delta G}_{{\rm{c}}}={\Delta }_{{\rm{m}}}{\dot{\gamma }}\left[{\Delta }_{{\rm{b}}}/{\Delta }_{{\rm{m}}}-{\mathrm{ln}}{\dot{\gamma}}\right]\end{array}$$

The total free energy ΔG_T_, including the newly proposed retained damage within the metastable solid, drives the subsequent transformation and results in a shorter incubation delay.

**Fig. 4:**
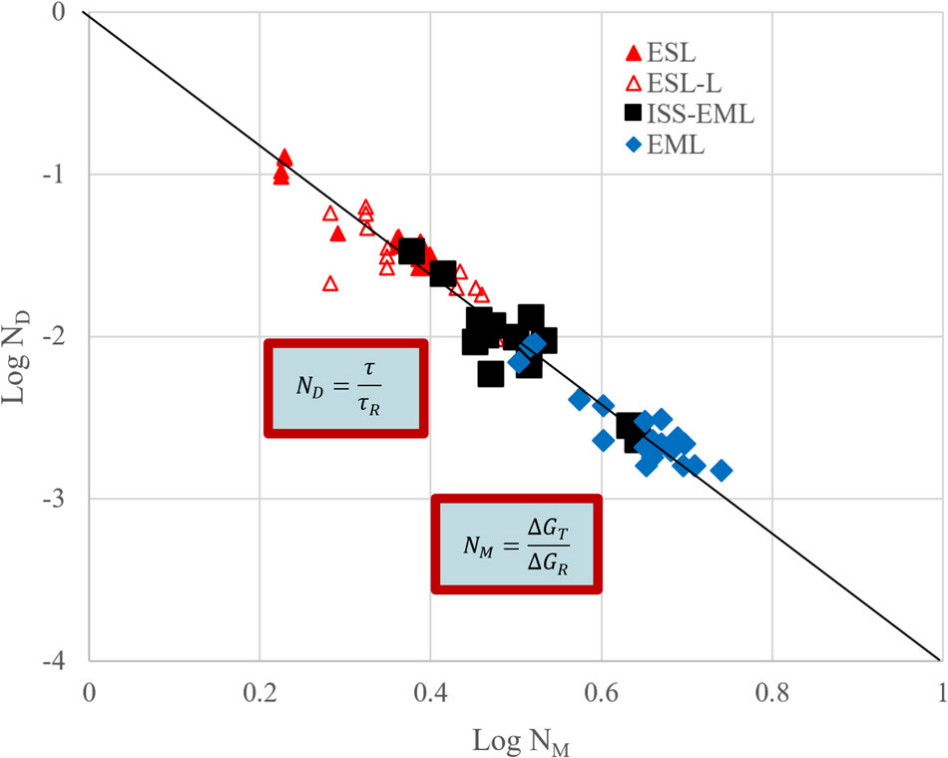
Retained damage model performance comparing dimensionless delay time to the dimensionless driving force.

The lines in Fig. 3b correspond to RDM predictions at the given shear conditions. These results can also be presented in a dimensionless form where the experimental results are normalized by a convenient reference condition that defines two new dimensionless groups, *N*_D_ = *τ/τ*_R_ and *N*_M_ = *ΔG*_T_*/ΔG*_R_ whose relationship captures the dependence of transformation delay on overall retained free energy. Predictions from the RDM are then presented in Fig. 4. The reference condition for normalizing delay and driving force is taken from an experimental determination of the maximum delay, which is observed with zero stirring and at the minimum undercooling required to access the metastable phase (and thus minimum driving force) as defined by the phase diagram. This condition is indicated in Fig. 3b as an orange star. The observed slope of the dimensionless log-log plot is negative 3.992 ± 0.027, which compares favorably with the theoretical value of negative 4.0 from Eq. 3.

3 $$\tau =\frac{128\,\pi \,{k}_{B}\,T\,{\gamma }^{3}f\left(\theta \right)}{\beta }{\left(\frac{\Omega }{{N}_{{\rm{A}}}}\right)}^{2}\left[{{\Delta G}_{{\rm{T}}}}^{-4}\right]$$

Here*, τ* is the delay, *k*_B_ the Boltzmann constant, *T* the transformation temperature, *γ* the surface energy, *f(θ)* the cluster geometry constant, *Ω* the molar volume, *N*_A_ Avogadro’s number, *β* the attachment frequency, and *ΔG*_T_ the total retained free energy from Eq. 2.

Since undercooling and convection are extremely important in the control of industrial processes such as die casting, investment casting, directional solidification, welding, and additive manufacturing, the new RDM prediction is a transformative advancement for use in modeling the production of high-quality, reliable products to optimize local properties and manage the development of defect structures both in space and in terrestrial, industrial applications.

### Peritectic alloy rapid solidification with electromagnetic convection (PARSEC)

The PARSEC program involves the investigation of metastable phase transformation by nucleation in the interdendritic liquid phase.

**Fig. 5:**
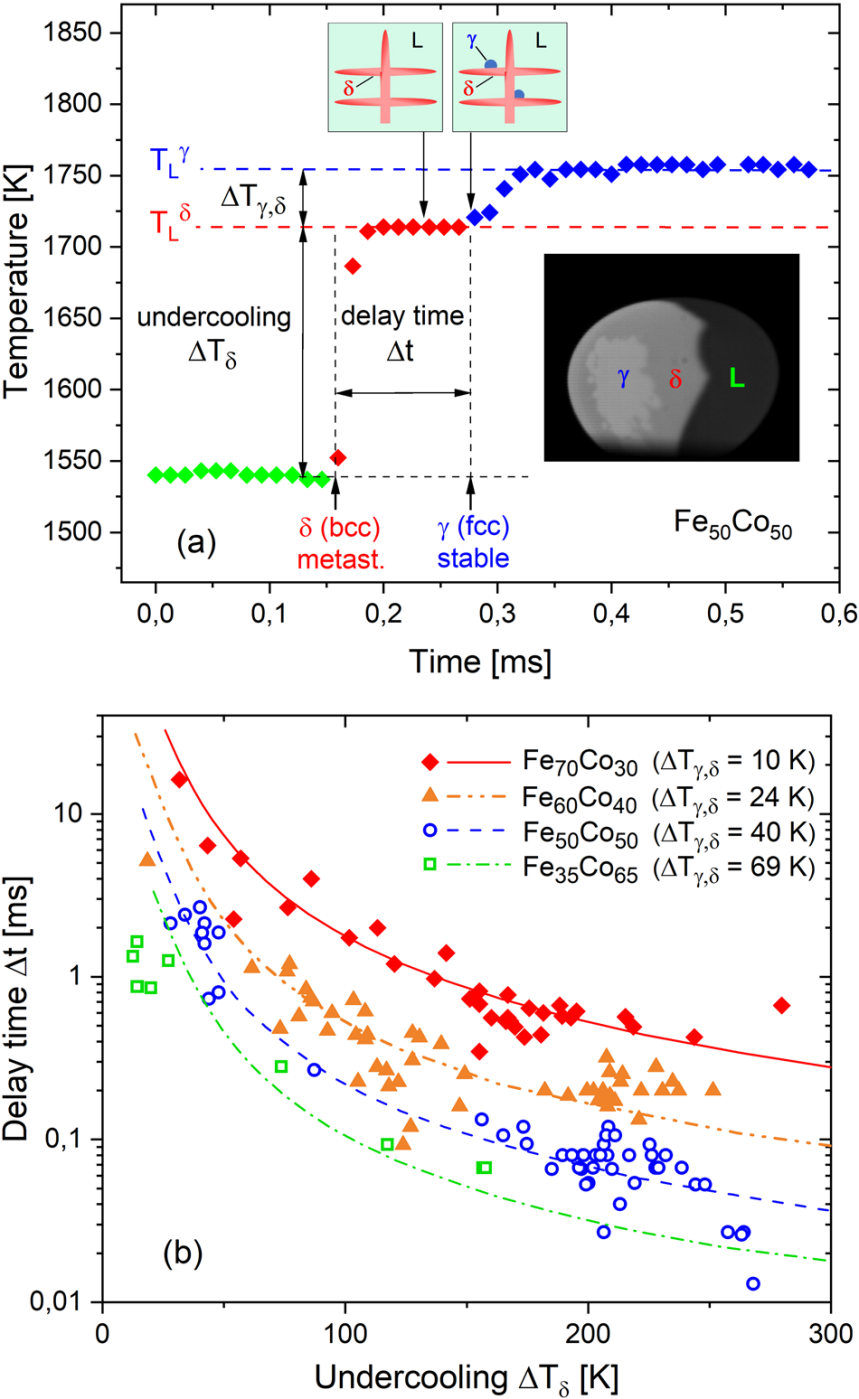
Rapid solidification of FeCo alloys. **a** Temperature-time profile taken at the nucleation point and high-speed video image (insert) during double recalescence with primary metastable primary $\delta$ phase solidification and subsequent transformation into stable $\gamma$ phase in ground-based electromagnetic levitation tests on Fe_50_Co_50_. Heterogeneous nucleation of stable $\gamma$ phase at primary dendrites of the metastable $\delta$ phase during the intermediate plateau is illustrated by the inserts at the top. **b** Measured delay times (symbols) as a function of the undercooling ${\Delta T}_{\delta }={T}_{{\rm{L}}}^{\delta }-{T}_{{\rm{N}}}$ for different Fe-Co alloys obtained by high-speed video analysis in comparison to model prediction (lines)^43^.

Generally, upon solidification of a metastable phase in the undercooled melt, the stable phase can nucleate either in the metastable solid or in the residual liquid phase within the mushy-zone. Metastable phase transformation will be dominated by the mechanism with the fastest kinetics, which may depend on the alloy system and the experiment conditions. As shown by the temperature-time profile in Fig. 5a, during rapid solidification of an electromagnetically levitated Fe_50_Co_50_ sample, the temperature is raised to an intermediate plateau; which is located near the liquidus temperature ${T}_{{\rm{L}}}^{\delta }$. Here, the sample consists of dendrites of primarily solidified metastable $\delta$ phase and interdendritic liquid phase (mushy-zone) as illustrated by the inserts in Fig. 5a. Hence, the interdendritic liquid is undercooled by an amount corresponding to the difference between liquidus temperatures of stable $\gamma$ and metastable $\delta$ phase, ${\Delta T}_{\gamma ,\delta }={T}_{{\rm{L}}}^{\gamma }-{T}_{{\rm{L}}}^{\delta }$. Upon the second recalescence step, due to nucleation and growth of the stable $\gamma$ phase, the temperature is further raised to the equilibrium liquidus temperature ${T}_{{\rm{L}}}^{\gamma }$ by which the primary dendrites are either remelted or transformed into the stable phase. A model for the delay time for the conversion from metastable $\delta$ to stable $\gamma$ phase considers the steady-state nucleation rate, ${I}^{s}={A}^{* }\exp \left(-{W}_{{\rm{het}}}^{* }/{k}_{{\rm{B}}}T\right)$, according to the classical nucleation theory and assumes heterogeneous nucleation of $\gamma$ phase in the liquid at the surface of pre-existing dendrites of metastable $\delta$ phase (see Fig. 5(a))^43,44^. The pre-factor ${A}^{* }={N}_{{\rm{het}}}\,\cdot\, K$ depends on the attachment rate of atoms to the nucleus, $K$, and the total number of potential heterogeneous nucleation sites, ${N}_{{\rm{het}}}$, which scales with the surface area of the dendritic network. Hence, with the expanding mushy-zone during the growth of the metastable phase, the number of nucleation sites ${N}_{{\rm{het}}}$ rises with time. As growth velocity is increased with increasing undercooling, the number of nucleation sites and, therefore, the nucleation rate are rising faster as well. In addition, the interface area between dendrites and residual melt becomes larger with rising undercooling, which is due to a finer dendrite thickness and an increased fraction of solid, thus leading to a higher number density of potential nucleation sites. Consequently, a critical nucleus of the stable phase is formed after a shorter period of time, i.e., the delay time is shortened as undercooling is raised. The evolution of the number of potential nucleation sites and, therefore, the heterogeneous nucleation rate over time is determined by the growth velocity $v$ and the interface area estimated by the dendrite tip radius $r$ of primary $\delta$ phase, which lead to the relation for the delay time^43^:

4 $$\Delta t \sim {\left(\frac{{\Delta H}_{{\rm{f}}}}{{c}_{{\rm{p}},{\rm{L}}}\,\cdot\, K}\right)}^{1/4}\,\cdot\, {\left(\frac{r}{{\Delta T}_{\delta }\,\cdot\, {v}^{3}}\right)}^{1/4}\,\cdot\, \exp \left(\,\frac{1}{4}\,\frac{{\Delta G}_{{\rm{het}}}^{* }}{{k}_{{\rm{B}}}{T}_{{\rm{L}}}^{\delta }}\right)$$

Here ${c}_{{\rm{p}},{\rm{L}}}$ is the specific heat of the liquid and ${\Delta H}_{{\rm{f}}}$ is the latent heat of fusion of $\delta$-phase. The exponential term depends on the activation energy ${\Delta G}_{{\rm{het}}}^{* }=\left(16\pi /3\right)\,\cdot\, {\sigma }_{{\rm{S}},{\rm{L}}}^{3}\,\cdot\, f/{\Delta G}_{{\rm{V}}}^{2}$ (${\sigma }_{{\rm{S}},{\rm{L}}}$: solid-liquid interfacial energy, $f$: catalytic potency factor, ${\Delta G}_{{\rm{V}}}$: Gibbs free energy difference per volume) for heterogeneous nucleation taken at the undercooling of the interdendritic, residual melt, ${\Delta T}_{\gamma ,\delta }={T}_{{\rm{L}}}^{\gamma }-{T}_{{\rm{L}}}^{\delta }$, and reflects the effect of alloy composition. Both growth velocity $v$ and tip radius $r$ are functions of the undercooling with respect to the liquidus temperature ${T}_{{\rm{L}}}^{\delta }$ of metastable $\delta$ phase, ${\Delta T}_{\delta }={T}_{{\rm{L}}}^{\delta }-{T}_{{\rm{N}}}$ (${T}_{{\rm{N}}}$: nucleation temperature of the undercooled melt) and are determined by models for dendritic growth in undercooled melts^45,46^.

As shown in Fig. 5b the measured delay times plotted versus ${\Delta T}_{\delta }$ are well described by the model in a wide range predicting an undercooling dependency according to ${({r/{\Delta T}_{\delta }v}^{3})}^{1/4}$. The nearly parallel curves are shifted towards shorter delay times with increasing Co concentration, which is mainly due to the larger temperature interval ${\Delta T}_{\gamma ,\delta }$ according to the phase diagram^47^ and, therefore, to an increased Gibbs free energy difference acting as the thermodynamic driving force for phase transformation. As growth kinetics and pattern formation are generally influenced by fluid flow^48^ the model also involves convection effects since the delay time explicitly depends on growth velocity and dendrite tip radius. In addition, fluid flow effects can originate from the influence on the nucleation rate in terms of the pre-factor ${A}^{* } \sim K$^27^ that is enhanced with increasing melt shear rate, as reported in the present article.

### Non-equilibrium solidification (NEQUISOL)

The Canadian collaborative effort within this ESA project involves Containerless Solidification and Characterization of Industrial Alloys. In particular, the group studies atomization and evaluation of the solidification path during powder production.

Impulse atomization (IA) is a drop tube-type containerless solidification technique where solidifying droplets experience high cooling rates and nucleation undercooling. It consists of a transformation of a bulk liquid into a spray of liquid droplets that solidify rapidly during free fall by losing heat to the selected surrounding gas (usually N_2_, Ar, or He). The alloy is melted in a crucible using an induction furnace. Atomization is achieved by the application of a mechanical pressure (impulse) to the melt in order to push it through a nozzle plate with one or several orifices of known size and geometry. A liquid ligament emanates from each orifice, which in turn breaks up into droplets due to Rayleigh-type instabilities. The solidified powders are then collected in a beaker at the bottom of the tower. A detailed description of the process can be found in ref. ^49^. While EML can process one sample at a time, IA generates a range of droplet sizes per run (typically 50–1000 μm), giving a range of cooling rates and undercoolings. The cooling rate is a function of both the droplet size and the atomization gas and can reach up to ~10^5^ Ks^−1^. Furthermore, solidification in free fall greatly minimizes convection within the samples. Thus, IA and EML are complimentary solidification techniques for the evaluation of rapidly solidified samples.

**Fig. 6:**
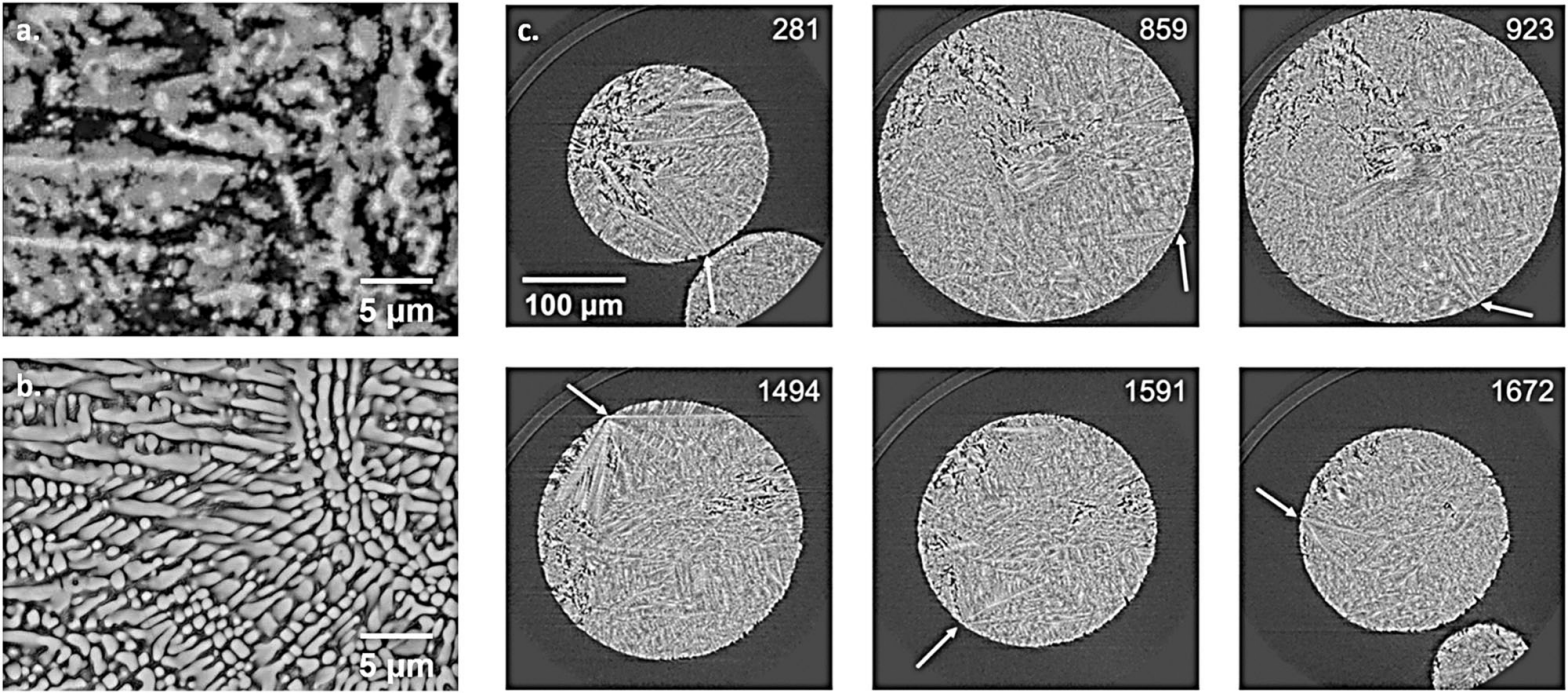
BSE-SEM micrographs of Al-36wt%Ni (Al_80_Ni_20_) particles atomized in helium. Panels show **a** diameters of 328 μm and **b** diameters of 150 μm; **c** Selected synchrotron x-ray microtomography slices of a 310 μm Al-36wt%Ni (Al_80_Ni_20_) droplet atomized in helium. The arrows denote nucleation points at the surface of the droplets, with primary dendrite arms growing towards the center of the droplet.

Figure 6a shows the microstructure of a 328 μm Al-36wt%Ni droplet atomized in helium. Solidification starts with the nucleation and growth of primary Al_3_Ni_2_ (light gray phase). Upon further cooling, a peritectic reaction occurs between the liquid and the primary phase to form Al_3_Ni (dark gray phase). Due to the rapid solidification conditions, the peritectic transformation is however incomplete. The core of the primary dendrites remains as Al_3_Ni_2_. Finally, the remaining liquid solidifies as an α-Al– Al_3_Ni eutectic (black areas). Some particles with diameters smaller than 180 μm exhibit a vastly different microstructure, as shown in Fig. 6b. The primary dendritic structure shows no sign of an incomplete peritectic reaction. Furthermore, compositional analysis indicates that the primary phase is actually Al_3_Ni. Along with its morphology, this suggests that the high cooling rate experienced by the smaller droplets suppresses the formation of Al_3_Ni_2_ and enables the direct nucleation of Al_3_Ni from the liquid. Upon reaching the eutectic temperature, the remaining liquid then solidifies as α-Al– Al_3_Ni. Neutron diffraction analysis of different droplet sizes also showed that the Al_3_Ni_2_ to Al_3_Ni decreases as the particle size decreases (i.e., as the cooling rate increases)^49^. Furthermore, it was found that particles with diameters smaller than 275 μm contain some peaks that correspond to the formation of a quasicrystalline phase, known as D-phase. Löser and Shuleshova showed that this metastable forms 925 to 975 K^50^, which corresponds to an undercooling of 150 to 200 K. Devred et al. found retained D-phase in gas-atomized particles with diameters smaller than 38 μm^51^. This indicates that the IA particles exhibiting peaks corresponding to the D-phase have experienced high primary phase undercooling and cooling rates during the solidification process. Figure 6c shows selected synchrotron x-ray microtomography slices of a 310-μm-diameter droplet atomized in helium. Similar to EML solidification in microgravity, several nucleation points are observed at the surface of the droplet, with dendrites growing toward the center of the sample.

**Fig. 7:**
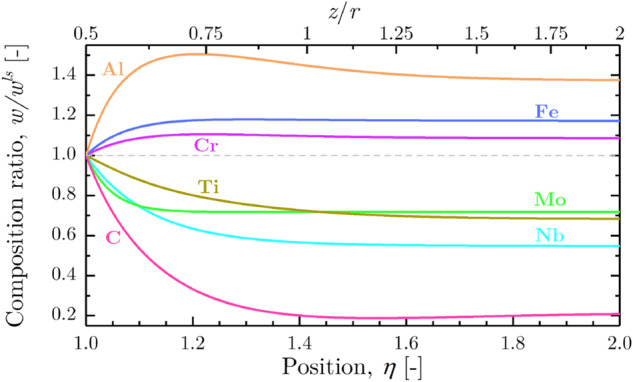
Ratio between local liquid composition at paraboloidal coordinate *η*, *w*(*η*), and liquid composition at the solid-liquid interface, *w*^*ls*^, for the alloying elements of the IN718 alloy, with velocity *v* = 0.3 m s^-1^. Coordinate *η* = 1 corresponds to the solid-liquid interface at the dendrite tip and coordinate *η* = 2 is located at a distance equal to 3*r*/2 from the tip of radius *r*^52^.

Modeling of the dendrite tip kinetics is key to understand the formation of microstructures in undercooled atomized systems. This is particularly true for multicomponent alloys for which only partial coupling with thermodynamic data was so far achieved. For this reason, a fully coupled dendrite tip kinetic model was recently developed and applied to a typical nickel-base alloy^52^. As shown in Fig. 7, the full diffusion profile of solute species is computed. It accounts for the full diffusion matrix in the liquid, hence explaining the existence of non-monotonous diffusion profiles^53^. Thermodynamic data are extracted from databases TCNI10^54^ and MOBNI5^55^. Comparisons with classical approximations are discussed, including linearization of the multicomponent phase diagram, diagonal diffusion, approximation of the curvature undercooling, and simplifications using multi-binary phase diagrams or a pseudo-binary phase diagram deduced from the former binary data. It is found that full coupling with thermodynamic data is essential for the application of theories to multicomponent alloys. Extensions to implement a direct assessment of kinetics undercooling is under developments^56^, opening the way to revisit the interpretation of some microgravity data.

### Non-equilibrium multi-phase transformation (MULTIPHAS)

The MULTIPHAS program targets the study of anomalous solidification kinetics from undercooled liquids.

**Fig. 8:**
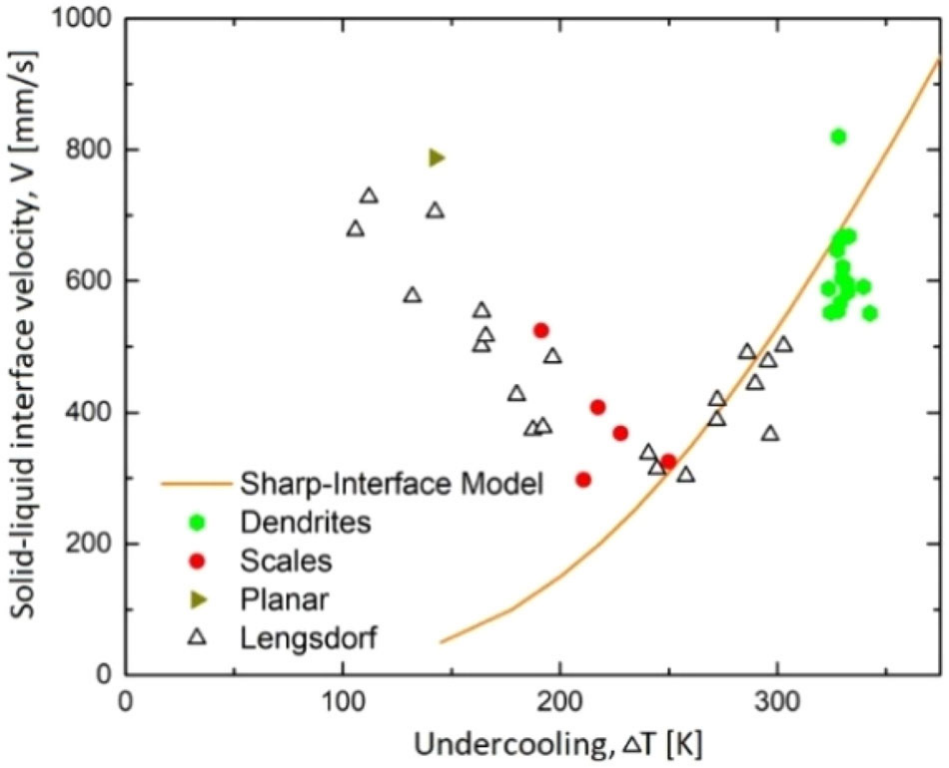
Front velocity versus undercooling of the Al-35at.%Ni alloy. The empty black triangles represent the previously obtained data by refs. ^58,59^. New data from the ISS-EML are marked with solid symbols, particularly a yellow triangle (planar), red circles (scales), and green hexagons (dendrites). The curve has been calculated with a sharp interface model^57^, showing that beyond ΔT = 250 K the expected velocity trend occurs. Calculations were made for the primary NiAl(B2)-phase. Experiments were performed on ISS-EML, flight data 08.02.2018, cycle 5; data points were found 15.05.2019 from 16 cycles ISS-EML.

**Fig. 9:**
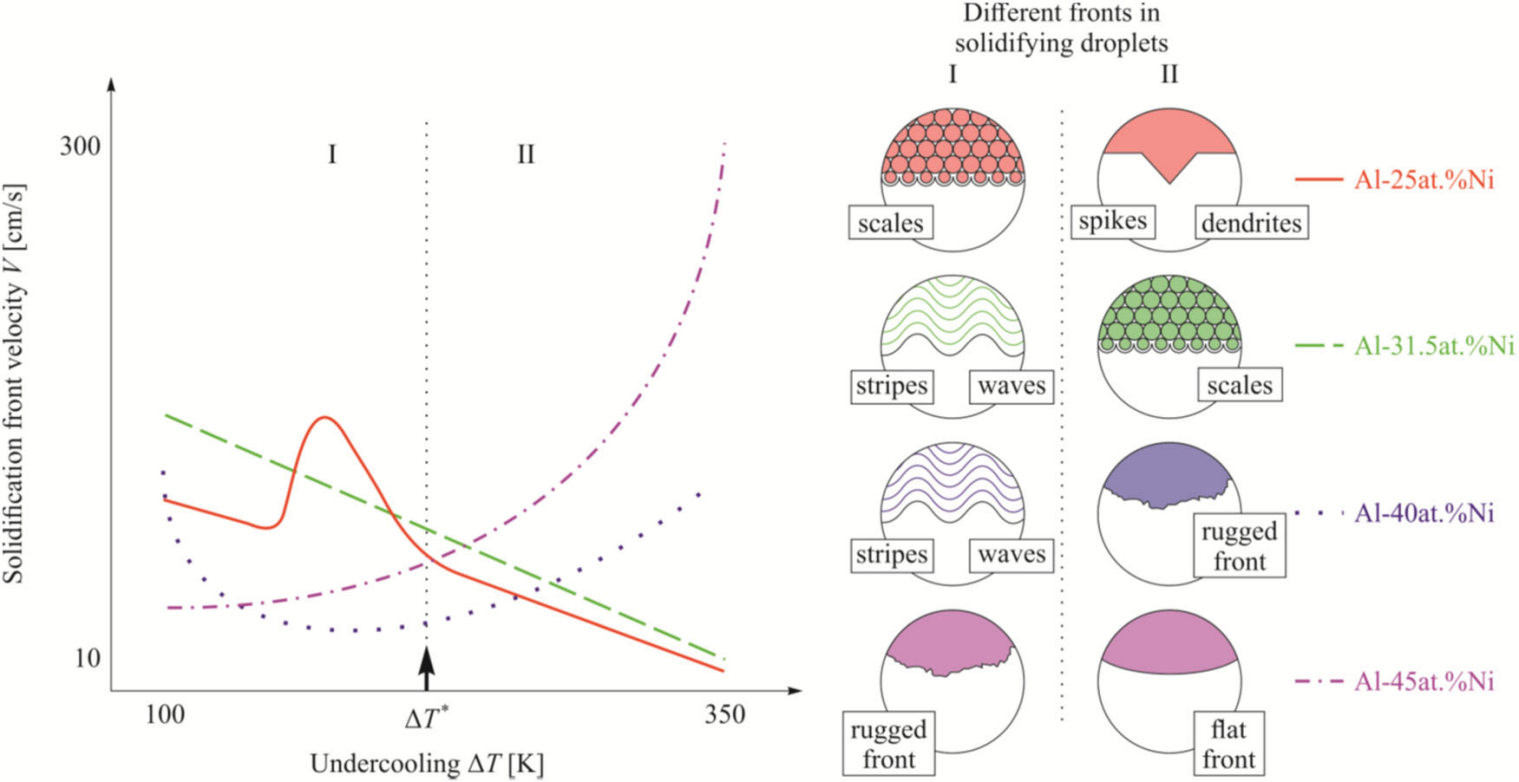
Classification of solidification morphologies observed on the surfaces of Al-Ni droplets processed in EML on the Ground, under reduced gravity (in parabolic flights), and in microgravity (onboard the ISS). Classification is given by the behavior of solidification front velocity *V* as a function of undercooling *ΔT*. Note that for the same undercooling, one can find different forms of recalescence and final primary microstructures depending of the content of Nickel, i.e., for different nominal compositions of the Al-rich Al-Ni alloys^61,62^.

From thermodynamical consideration, a monotonically increasing crystal growth velocity with increasing undercooling is expected in the crystallization of liquids, mixtures, and alloys^57^. By contrast to this general theoretical statement, Al-rich Al-Ni alloys show an anomalous solidification behavior in the “velocity versus undercooling” relationship^58,59^: at low undercoolings, Δ*T* < 250 K, the velocity unexpectedly decreases with increasing undercooling. For higher undercoolings, Δ*T* > 250 K, the crystal velocity increases with increasing undercooling following the thermodynamically consistent trend seen in Fig. 8. In the light of measurements in microgravity with an Al-25at.% Ni alloy sample onboard the ISS, results confirm this anomalous behavior as an unexpected trend in solidification kinetics. It is also found that besides the anomalous growth behavior, changes in the morphology, or recalescence growth front, occur. Indeed, the experimental results obtained using a space high-speed camera coupled with subsequent metallographic analysis upon sample return have been compared with previous data gained on the ground^58,59^ using theoretical approaches based on statistical and analytical techniques. The experiments show different growth front morphologies in defined distinct ranges of undercoolings: planar front, scales, and dendrites, as displayed in Fig. 9. Scales are observed in the undercooling range where anomalous growth behavior is observed, i.e., for decreasing growth velocities with increasing undercooling. The scaled front consists of numerous nuclei ahead of the crystal-liquid interface that occurs in the undercooling range featuring the negative slope for the crystal growth velocity at Δ*T* < 250 K. This unusual behavior is confirmed experimentally and explained theoretically using analytical solutions for the crystal growth front with the polydisperse ensemble of crystals nucleating ahead of the parent front^60,61^.

**Fig. 10:**
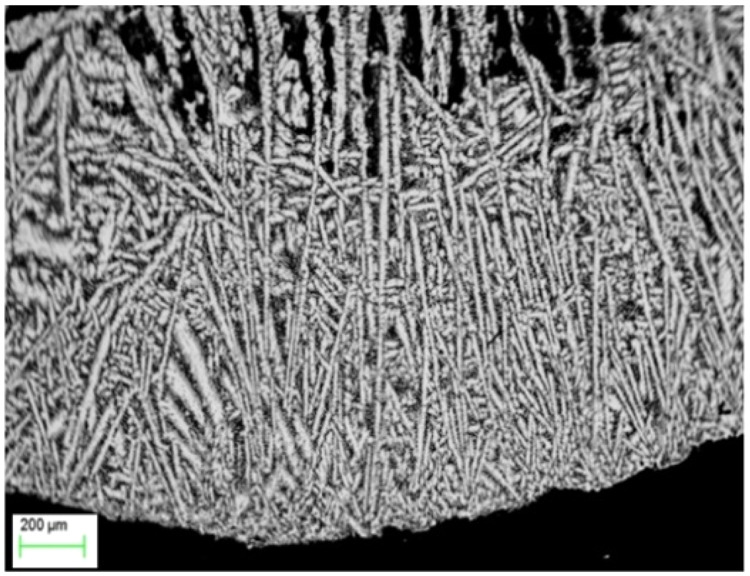
Image of the microstructure from a sample of Al-35at.%Ni alloy solidified with an undercooling of *ΔT* = 189 K. This cross-section shows dendrites that are growing inwards toward the center of the sample, which is located at the top of the micrograph.

The scale distribution was analysed, and the results show that a lower number of larger scales forms with increasing undercooling. The analysis method for determining the front of recalescence (as an envelope of the crystallization front)^58,59^ remains also appropriate for this front morphology, however, the observed front represents a predominantly nucleation front successfully competing with the growth front of dendrites^61^. This is confirmed by the in situ observation of the recalescence front using the high-speed camera as well as by metallographic evaluations on images of the microstructures shown in Fig. 10.

The measurements show multiple nucleation events forming the growth front in Fig. 10, confirming a mechanism that has been observed for the first time in Al-Ni alloys^60,61^ over the wide range of concentrations shown in Fig. 9. In particular, the experimental growth measurements and metallographic data directly demonstrate that the growth front does thus not consist of dendrite tips (as in usual rapid solidifying samples), but of newly forming nuclei propagating along the sample surface in a coordinated manner. Therefore, the measured velocity at small undercooling, Δ*T* < 250 K, is related to the nucleation events but not to the growth phenomena^60,62^.

### Chill cooling for the electromagnetic levitator in relation to the continuous casting of steel (CCEMLCC)

The CCEMLCC program investigates directional solidification from levitated steel samples utilizing a quench system based on chill-casting the sample through contact with an inert substrate.

Solidification processes are generally accompanied by liquid flow and thermomechanical deformations originating from the density difference between the liquid and the solid states as well as from thermal contraction^63^. As a benchmark experiment, deformation due to shrinkage is investigated during directional solidification of levitated droplets of different steel alloys in the frame of the European Space Agency (ESA) project entitled Chill Cooling for the ElectroMagnetic Levitator in relation to Continuous Casting of steels (CCEMLCC). A quenching device was developed, tested, and implemented for chill solidification of levitated steel droplets for use in the ISS-EML facility.

A Fe-0.9C-0.2Si (wt.%) steel droplet with an average diameter of 6 mm, was processed in the ISS such that the first few cycles were dedicated to thermophysical property measurements carried out in collaboration with the team of the THERMOLAB project at the University of Ulm. They included liquid viscosity and surface tension as a function of temperature—both properties are key inputs for modeling activities relating to flow simulation within the mushy-zone.

**Fig. 11:**
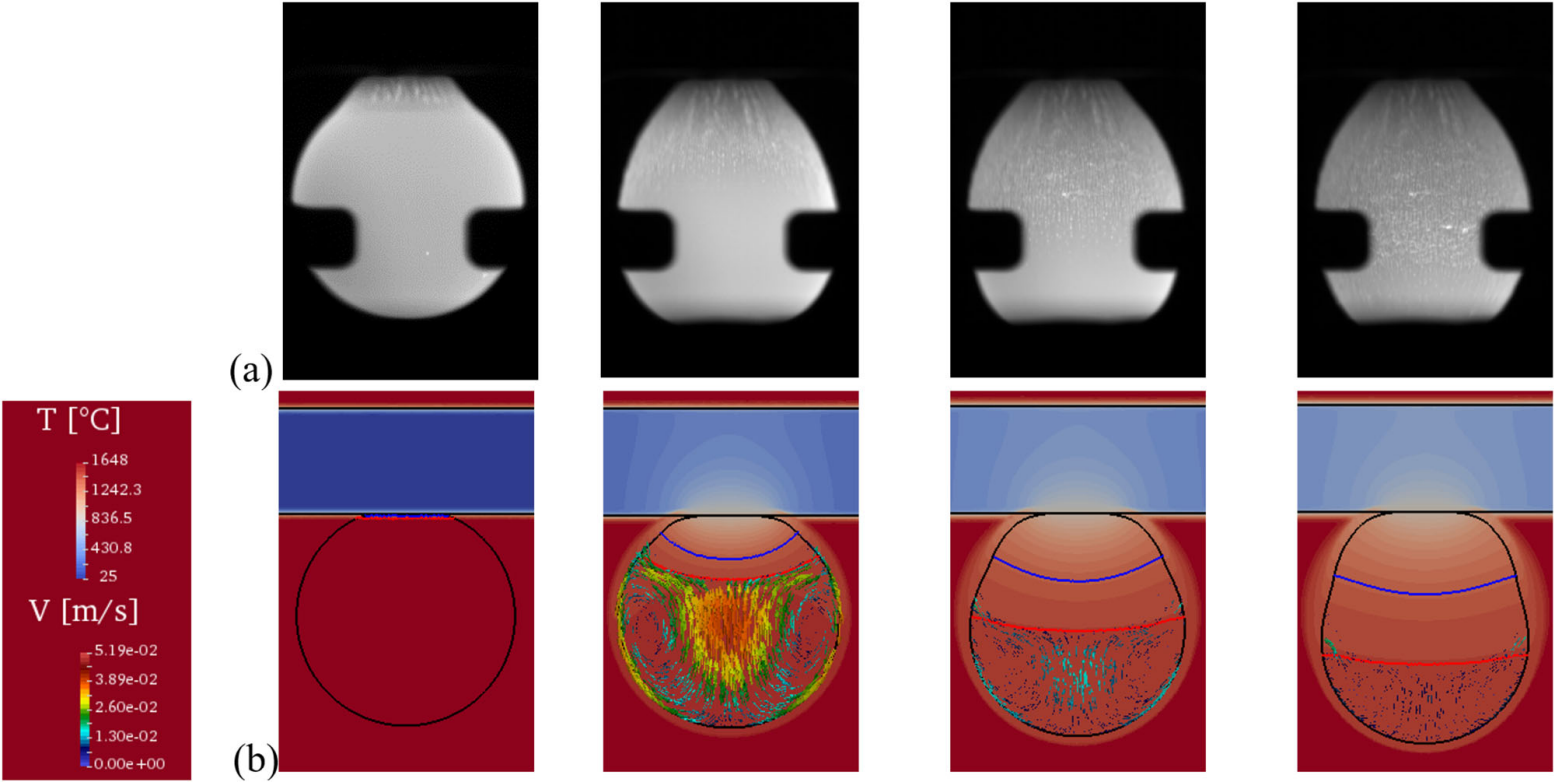
Evolution of structure during chill casting. Sequence of **a** video images during chill cooling of the Fe-0.9C-0.2Si (wt.%) sample (ID#20, Batch#2, ISS) with an average diameter of 6 mm and **b** thermohydraulic simulation with (round black contour) free liquid-gas boundary showing the temperature field in the (cold colors) cooling device, (hot colors) the metallic droplet and the surrounding gas, as well as (colored arrows) the velocity field and (red line) the start and (blue line) the end of solidification. Sample provided courtesy of ArcelorMittal (Maizières-lès-Metz, France).

The final cycle was dedicated to chill cooling. By contacting the levitating liquid droplet with a Si_3_N_4_ plate positioned at one end of the sample cup holder, directional solidification took place. The long-term microgravity environment on ISS allowed complete solidification of the entire droplet under ideal and controlled conditions without external forces, which was not possible with parabolic flights. Figure 11a shows the time evolution of the droplet shape during its solidification. The sample progressively elongates, starting from its initial equilibrium spherical shape and finally adopting a deformed shape. Comparisons with previous parabolic flight campaigns show that this behavior is presumably dominated by several parameters such as alloy composition, type of solidifying phase (ferrite or austenite), solidification interval between liquidus and solidus temperature, and partitioning behavior of alloying elements. Samples processed in the ISS are expected to be returned to the ground soon for inspections, including metallography, optical imaging, x-ray radiography, neutron diffraction, and microprobe analyses. Meanwhile, modeling of both temperature and fluid flow velocity field during chill cooling have been carried out as shown in Fig. 11b. The numerical simulation includes a thermohydraulic solution with surface tension and Marangoni convection, also accounting for solute mass and total mass conservations with shrinkage flow, the metal-gas boundary being dealt with a level set formulation^64,65^.

**Fig. 12:**
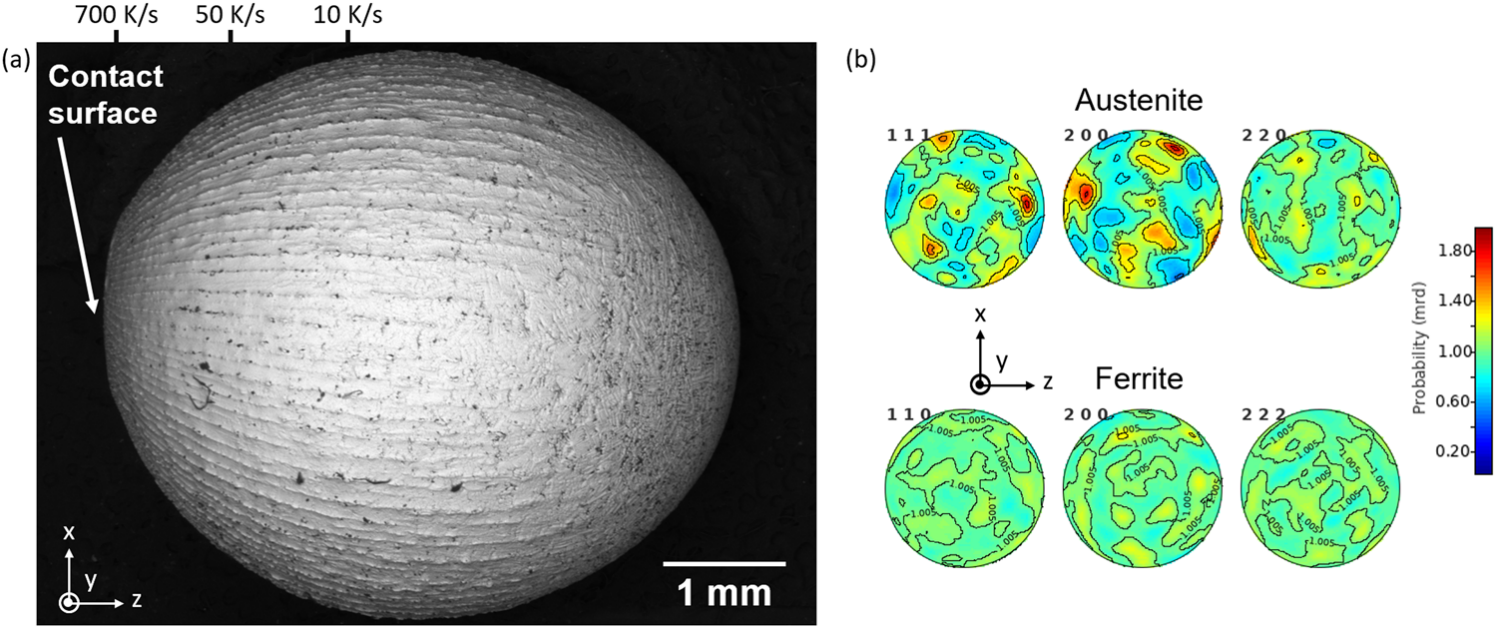
Structural characterization of a chill-cooled D2 droplet. **a** SEM imaging of the surface; columnar dendrites are propagating away from the contact surface and a columnar to equiaxed transition is observed. **b** Pole figures obtained with neutron diffraction over the whole sample. Austenite has a stronger texture than ferrite.

Solidification of D2 steel, Fe-1.55C-11.8Cr-0.40Mn-0.80Mo-0.80V-0.30Si (wt%), was also investigated. Figure 12 (left) shows a scanning electron microscope image of a sample processed in microgravity during a parabolic flight. Solidification started at the contact surface between the sample and the quenching plate. An array of columnar dendrites propagates away from the contact surface, covering about three quarters of the sample. Then, a columnar to equiaxed transition is observed, leading to the remaining part of the sample (on the opposite end of the quench plate) solidifying as equiaxed dendrites. Cooling rates are estimated from the measurement of the secondary dendrite arm spacing at various locations along the sample. A strong deformation of the initially spherical droplet is also observed along the direction of heat extraction (normal to the chill plate). D2 Tool Steels are ferritic at room temperature. However, due to the high carbon content, the first phase to form upon solidification is austenite. Previous studies on this alloy showed that rapid solidification can suppress the transformation from austenite to ferrite. Droplets generated by Impulse Atomization (102–105 K/s) were fully austenitic^66^, while samples solidified using EML (10–50 Ks^−1^) showed 5–6% ferrite^67^. Neutron diffraction was carried out at Los Alamos National Laboratory (LANL) on the chill-cooled sample shown in Fig. 12. Results of Rietveld analysis of the diffraction spectra yielded an average of about 56% of austenite and 44% of ferrite over the whole sample. Local measurements at both ends of the sample showed that the fraction of ferrite is lower, close to the contact surface. Furthermore, neutron results showed strong variations of measured and fit peak intensities as a function of sample direction, indicating that the sample is textured. This is confirmed by the pole figures obtained after further analysis of the bulk diffraction data. A strong texture is observed in austenite (red poles), while ferrite is random. This indicates that the columnar dendrites are mostly comprised of retained austenite, while the equiaxed dendrites should mostly be ferrite. This is also coherent with the heat extraction process during solidification. The cooling rate is highest at the contact surface with the quench plate and then decreases away from the plate. Thus, it is expected that the microstructure is composed of retained austenite at the contact surface. The fraction of transformed ferrite should then increase as the local cooling rate decreases.

### Liquid phase separation in metallic alloys (LIPHASE)

The LIPHASE project focuses on the demixing of undercooled melts.

**Fig. 13:**
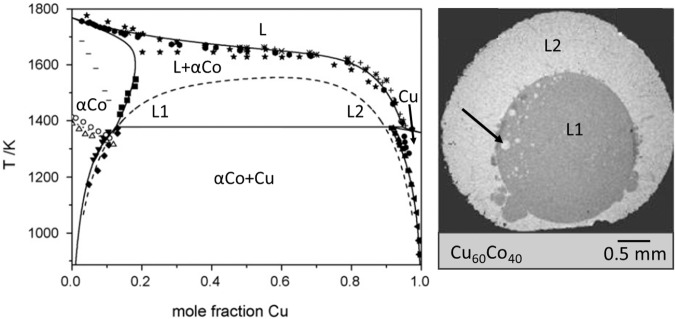
Miscibility gap in the Cu-Co system. Left: The Co-Cu phase diagram optimized by CALPHAD; the metastable miscibility gap in the liquid is given by the binodal (dashed line), which separates the regions of homogeneous liquid L and metastable liquids L1 and L2. When the homogeneous liquid L is undercooled into the metastable miscibility gap, L demixes into the metastable liquids L1 and L2 with their concentrations given by the binodal and the actual temperature. Solid lines indicate stable phase boundaries. Data points are experimental points from the literature, the sources are given in ref. ^68^. L, αCo, and Cu indicate the respective phases of the stable phase diagram. The dotted line in the αCo-region indicates the magnetic phase transition. Right: Cross section of a Cu_60_Co_40_ sample processed using differential scanning calorimetry (DSC). Backscattered electron image where dark regions are Co-rich and bright regions are Cu-rich representing the metastable L1 and L2 liquids. The arrow indicates a Cu-rich droplet within the solidified L1 phase.

Monotectic alloys exhibit demixing of the melt into two liquids—L1 and L2. In some Cu alloys of industrial interest (examples are Co-Cu-Fe and Co-Cu shown in Fig. 13), the miscibility gap occurs below the liquidus as revealed by the shape of the phase diagram^68^. It is accessed by deeply undercooling the melt. In experiments, the metastable solvus is reached by rapid solidification in containerless levitation processing on Earth or in microgravity^69,70^, in free fall in a drop tube^71^, by glass flux in a crucible^72^ or by chill block quenching^73^.

**Fig. 14:**
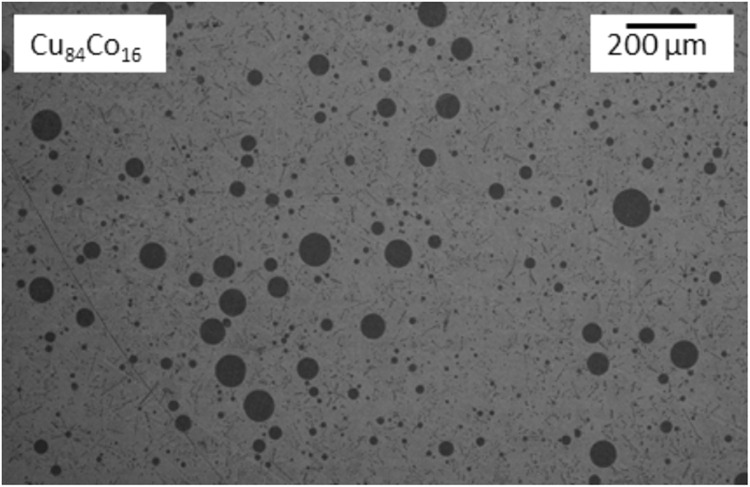
Dispersion of Co-rich L1-droplets in the Cu-rich matrix L2. The alloy has been undercooled and solidified during parabolic flight in low gravity.

Due to high melt undercooling, the demixed liquid is solidified rapidly and the microstructure provides information about the metastable states of demixing. Cu-rich alloys have been extensively investigated to study the development of a dispersion of Co-rich liquid droplets seen in Fig. 14. The degree of melt convection has a strong influence on the dispersions, shown by experiments under different levels of convection in high magnetic field^74^, during sounding rocket/parabolic flight or in terrestrial EML^71^. The release of latent heat by the Co-rich droplets upon solidification allowed observation of their movement in the melt in situ and thus allowed direct comparison of experimentally observed motion and predicted melt flow patterns using MHD modeling^75^.

Reducing the convection while cooling the melt slowly onboard the ISS should allow the formation of a single drop of L1 surrounded by the L2 matrix, both spherical. Analysing the oscillations of the inner droplet, the liquid–liquid interfacial tension is measured, which is a relevant parameter for the nucleation of L1 from the melt^70,71^. Preliminary experiments onboard the TEXUS 44 rocket have provided values of the melt surface tension in agreement with previous data (1.21 Nm^−1^) and a first estimate of the L1-L2 interfacial tension (0.17 Nm^−1^ at 1863 K)^70^.

The demixing was studied in mm-sized spheres of Co-Cu alloys of various compositions embedded in an oxide glass which were heated to the liquid state and cooled at a constant rate directly into the cell of a DSC apparatus (inset of Fig. 13)^76^. Using the liquid–liquid interfacial free energy and the bulk free energy difference between L1 and L2 obtained by means of a new CALPHAD assessment of the Co-Cu phase diagram^68^ to model the nucleation of phases, it has been established that the liquid–liquid separation is nucleation controlled and occurs without detectable undercooling below the binodal line, contrary to the nucleation of the Co-rich crystal phase which is undercooled more than three hundred degrees below the liquidus.

The measurement of the interfacial tension as a function of temperature requires additional future experiments on the ISS. Compared to a sounding rocket, which provides a microgravity time span of a few minutes, ISS experiments might be extended to a longer duration. In this case, the formation of a large Co-rich droplet in a Cu-rich shell might be accessible - which is the starting point for measurement of the interfacial tension.

**Fig. 15:**
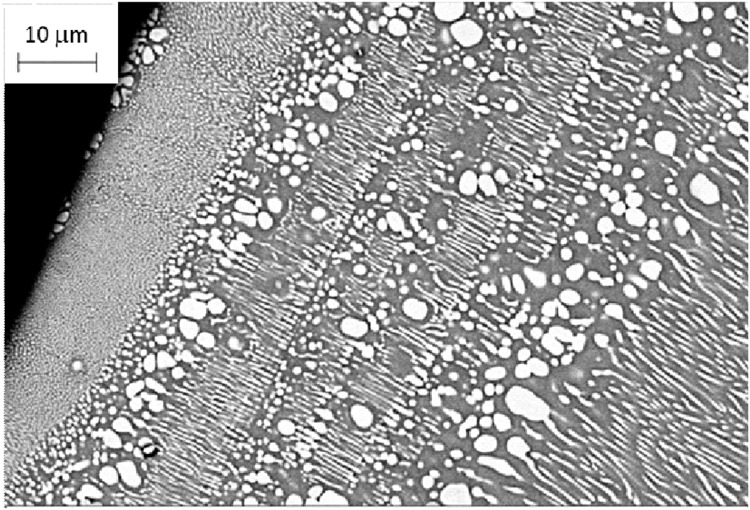
Regular/anomalous eutectic bands in a mold cast cylinder of 2 mm dia Co-61.8at%Si alloy.

Eutectic alloys solidify by demixing the homogeneous melt into two solid phases. Near the thermodynamical equilibrium, the two phases grow co-operatively, leading to a regular lamellar structure. The solidification melts in the undercooling regime generally produce anomalous eutectic structures with the outgrowth of a phase either in the form of dendrites or faceted crystals. These microstructures are influenced by convection. The phenomenon was investigated by means of various containerless processing techniques: electromagnetic levitation (EML), electromagnetic levitation under a static magnetic field (EML + SMF), and electrostatic levitation (ESL) employing the model line compound-line compound CoSi-CoSi_2_ eutectic^77,78^. Varied microstructures were achieved as a function of increasing undercooling occurring in the diverse techniques: primary dendrites or cells with regular eutectic, anomalous eutectic made of primary CoSi_2_ with intergranular CoSi, complex shape of the primary phase embedding precipitates. The pathways to microstructure formation in EML samples as a function of undercooling were determined by X-ray diffraction^79,80^, including and evaluation of remelting behavior^81^. Further aspects of the solidification were revealed by both melt spinning (MS) and copper mold casting (CMC)^82^ The first phase nucleating heterogeneously on the surface of the chilling block was most often the CoSi compound, as seen in Fig. 15, and then eutectic microstructure began to appear, however, in the adjacent melt more CoSi crystals were nucleated independently giving rise to an anomalous eutectic microstructure, with no coupled growth. Then a rod-like eutectic appeared, followed by a transition to a zone where primary CoSi crystals were formed again by re-nucleation.

This banded microstructure was repeated four times before the regular rod-like eutectic filled the alloy. It is apparent that fluctuations in growth rate occur, producing bands of the two types of eutectic. This stems from oscillations in the level of undercooling caused by the interplay of heat subtraction and recalescence. Melt-spun ribbons confirmed the scenario in smaller samples. The microstructural features (eutectic spacing and bandwidth) allow estimating the growth rates according to the Han-Trivedi model of band formation^83^.

The directional heat extraction implied by copper mold casting and melt spinning apparently has overcome the convection and allowed to show the banding in the CoSi-CoSi_2_ eutectic. The microstructure found in directional solidification has also a practical counterpart for repairing processes. Microgravity experiments will be very relevant to gain a better understanding of the phenomenon.

## Outlook and summary

To produce materials that meet ever-higher specific requirements and performance, the solidification processing of materials has to be controlled with ever-increasing precision. It is foreseen that the materials of tomorrow will be optimized in their design, featuring tailored composition for their specific application, underpinning more efficient production conditions, enhancing utilization of scarce resources, and favoring cleaner and more sustainable processes. At the heart of these initiatives is an understanding that structural evolution during liquid-solid phase transition requires experimental validation of theoretical and applied models across a broad range of length scales.

Moving forward, developments in alloy design and manufacturing process control are increasingly directed by numerical modeling and simulation—heading eventually to integrated computational materials engineering techniques. Besides accessing a robust material property database (which includes not only an understanding of measurement accuracy but also a statistically-based reporting of precision to allow quantification of reliability and performance variability), advancing state of the art in model development requires a knowledge of how convection influences the coupled processes involved in momentum transport, heat transfer, and mass transfer with a focus on interfacial phenomena.

One major thrust will be advancing the development of process modeling capability supporting additive manufacturing with an eye toward developing in-space manufacturing, material reuse and recycling, and in situ resource utilization capabilities to support the human exploration and colonization missions off-planet. Low earth orbit (LEO) offers a unique environment where surface tension-driven or Marangoni, the flow may be studied by eliminating buoyancy and sedimentation effects and where sample exchange and sample return is readily accomplished. The ISS-EML facility allows for the selection of the desired stirring conditions and provides an enabling and powerful tool for the advancement of knowledge in solidification physics across a wide range of experimental objectives and a diverse set of current and future metallic material classes.

One of the repeating themes from all previous discussions is the need to control convection. Here, we utilize the unique attributes afforded by ISS-EML processing to decouple the levitation and heating functions and then use the heating field to select the desired stirring conditions for a given experiment. Sometimes this is done to select the flow regime over the range from laminar to turbulent flow; sometimes to minimize stirring; but in all cases, to use convection as a quantifiable, controlled experimental parameter. As shown in Fig. 3a, magnetohydrodynamic modeling is used to evaluate stirring and melt shear as a function of experimental conditions and control convection to desired levels^84–87^. Differential evaporation from complex industrial alloys can also be monitored over the test duration to set experimental conditions to minimize the potential for unwanted compositional shifts for volatile components in the melt^88^. These models require an understanding of the melt thermophysical properties—a subject of a sister article as part of this ESA review series. One additional significant advantage of the ISS-EML facility is the ability to measure thermophysical properties in parallel with measurements on rapid solidification physics^89,90^.

### Reporting summary

Further information on research design is available in the Nature Research Reporting Summary linked to this article.

### Supplementary information

Reporting Summary

## Data Availability

All data were available through contact with the authors.
